# Risk factors for diabetic foot ulcers mortality and novel negative pressure combined with platelet-rich plasma therapy in the treatment of diabetic foot ulcers

**DOI:** 10.3389/fphar.2022.1051299

**Published:** 2022-12-16

**Authors:** Yanling Wang, Bang Liu, Yinzhen Pi, Li Hu, Yeling Yuan, Jiao Luo, Yixiao Tao, Ping Li, Shan Lu, Wei Song

**Affiliations:** ^1^ Changsha Maternal and Child Health Hospital Affiliated to Hunan Normal University, Changsha, China; ^2^ The First Hospital of Changsha, Changsha, China; ^3^ Department of Pediatrics, The Seventh Affiliated Hospital, Sun Yat-sen University, Shenzhen, China; ^4^ National & Local Joint Engineering Research Center of High-Throughput Drug Screening Technology, College of Health Science and Engineering, Hubei University, Wuhan, China

**Keywords:** diabetic foot ulcers (DFUs), diabetic patients without foot ulceration (NFU), negative pressure wound therapy (NPWT), platelet-rich plasma-fibrin glue (PRP), mortality

## Abstract

The purpose of this study was to assess the risk factors for morbidity and mortality of diabetic foot ulcers (DFUs). For the treatment of diabetic foot ulcers, negative pressure wound therapy (NPWT) combined with platelet-rich plasma-fibrin glue (PRP) was also investigated. There were 653 patients in the diabetic foot ulcer group and 510 patients in the diabetic patients without foot ulceration (NFU) group, for a total of 1163 patients in the study samples after individuals without follow-up were excluded. The patients were randomized into two groups: the negative pressure wound therapy group and the negative pressure wound therapy combined with the PRP group. The findings of the univariate analysis revealed the blood indicators for predicting diabetic foot ulcer morbidity risk factors, such as C-reactive protein, albumin, creatinine, alkaline phosphatase, procalcitonin, platelets, 25-hydroxyvitamin D, β-2-microglobulin, monocyte ratio, low-density protein cholesterol (LDL), triglyceride, alanine aminotransferase (ALT), aminotransferase (AST), creatine kinase (CK) and total cholesterol. Using logistic regression analysis revealed only albumin and age to be independent predictors of diabetic foot ulcer mortality. Our study also revealed that, compared to negative pressure wound therapy alone, negative pressure wound therapy combined with PRP accelerated wound healing and reduced the mortality rate. According to the findings of this pilot study, new risk factors for diabetic foot ulcer morbidity and mortality have been found, and negative pressure wound therapy combined with PRP therapy may provide the first information that it is an effective adjunct treatment for diabetic foot ulcers.

## 1 Introduction

Foot ulcers are a devastating complication in the intermediate and advanced phases of diabetes, referring to a foot infection and deep tissue degradation caused by aberrant nerves and vascular lesions in the distal lower limbs (Lin et al., 2020; Monteiro-Soares et al., 2020; Reardon et al., 2020; Tabanjeh et al., 2020). According to annual data from the World Health Organization (WHO), more than 2 million people undergo amputation worldwide. Every 20 s, a lower extremity is amputated due to diabetic foot ulcers (DFUs) (Armstrong et al., 2017). Furthermore, ulceration prevalence rates varied from 19% to 34% depending on the investigated diabetes cohort and country (Armstrong et al., 2017). The mortality rates for DFUs were approximately 42% within 5 years (Everett and Mathioudakis, 2018). Due to their high rates of disability and mortality, DFU has become a major contributor to the leading threats to human health. As a result, determining how to prevent and treat DFUs has become a pressing issue that requires substantial attention and in-depth investigation.

Foot ulceration is a preventable ailment in which modest interventions can minimize amputations and death by as much as 70% through programs that lower risk factors (Krishnan et al., 2008). Identifying the role of risk factors contributing to this condition will enable health providers to develop more effective prevention programs, which could improve patients’ quality of life.

The main principles of treatment for DFUs are glycemic control, debridement, nutritional support, and wound infection control (Lim et al., 2017). It is essential to prevent infection of the wound and facilitate wound healing in DFU patients. Negative pressure wound therapy (NPWT) is beneficial for accelerating wound healing and managing wound infection by using the application of air-tight occlusive dressings and local sub-atmospheric pressure (Huang et al., 2014). Studies have demonstrated the efficacy of NPWT for the wound healing of DFU (Liu et al., 2018; Seidel et al., 2020; Campitiello et al., 2021). Moreover, platelet-rich plasma-fibrin glue (PRP) is an autologous blood product rich in growth factors and cytokines. PRP alone has been demonstrated to have a regenerative effect on wounds (Sommeling et al., 2013), burns (Venter et al., 2016), alopecia (Cervantes et al., 2018), osteoarthritis (Ye et al., 2018), and nerves (Ikumi et al., 2018). In addition, platelet-rich plasma can speed wound healing by inhibiting infection and biofilm formation. PRP is a promising method for treating DFUs (Smith et al., 2020). However, the efficacy of NPWT combined with PRP in treated DFU is still unknown.

The current study aimed to (1) explore potential risk factors for the morbidity and mortality of DFUs; and (2) investigate the efficacy of NPWT combined with PRP in the treatment of DFUs.

## 2 Materials and methods

### 2.1 Ethics approval and consent to participate

This study was approved by the Ethics Committee of The First Hospital of Changsha. The reporting of this study conforms to the STROBE guidelines. Written informed consent was obtained from all participants, and their privacy was protected. In this study, all data were collected by the Hospital Information System of The First Hospital of Changsha.

### 2.2 Study population

The research was carried out between February 2014 and February 2021. Patients with type I or type II diabetes participated in this study. There were two groups: one was the DFU group, and the other was the diabetic patients without foot ulceration (NFU) group. The patients without follow-up data were excluded.

We gathered the treatment outcome of patients with DFU to investigate the efficacy of NPWT combined with PRP in treated non-healing DFU. Inclusion criteria included the following: (1) diabetic patients with foot ulceration; (2) foot ulcers with a size not less than 10 cm^2^; (3) imaging data were approved; and (4) DFU was diagnosed with Wagner3 or > Wagner3 on the Wagner classification system (Li, 2021).

### 2.3 Data collection

The demographic information and blood samples were collected from 510 patients with NFU and 653 patients with DFU in the study. During the cross-sectional study, the nursing staff of the institutes carried out standardized face-to-face questionnaires (validated by the authority) to gather demographic and other health-related data for each participant. Follow-up data, including outcomes for patients with DFU, were obtained in August 2022 through a telephone interview with the patient or their family member.

The patients were randomized into two groups, the NPWT alone group and the NPWT combined with the PRP group between May 2020 and May 2021.

### 2.4 Treatment

Combined group: All patients in the combined group were subjected to debridement, NPWT for 5–7 days, and PRP twice for 14 days. Completely healed or with signs of infection, PRP can be washed off and discontinued at any time.

Control group: All patients in the control group were subjected to debridement, NPWT for 5–7 days, and conventional dressing, such as iodine–alcohol, until wound healing.

The guiding principles were as follows: the wound was initially treated with normal saline and prepared for debridement to remove dead tissues and hyperkeratotic skin. In addition, a second wash with normal saline was performed to remove any remaining particles. As soon as the wound beds were free of obvious necrotic tissue and purulent discharge, NPWT was delivered on days 5–7. Following the application of PRP or normal dressing, the wound was covered with paraffin gauze.

### 2.5 Preparation of PRP

In the clean room of the Endocrinology and Metabolism department of the First Hospital of Changsha, PRP was prepared by centrifuging 10-fold volume of the wound’s blood at 2,000 g for 10 min to get rid of the red blood cells and plasma. The material was then purified by repeating the preceding steps. On the day of the experiment, samples were taken on-site and used immediately.

### 2.6 Statistical analysis

SPSS Statistics version 26 (IBM Corporation, Armonk, New York, NY) was used for statistical analysis. Statistical analysis was performed by adopting StatView (SAS Institute, Cary, NC) and Statbox Pro 6.0 (GrimmerSoft, Issy-les-Moulineaux, France). The normality of the distribution of each quantitative parameter was assessed by conducting the Kolmogorov–Smirnov test. A univariate analysis was carried out by using the Student t-test to find significant differences between two groups in normally distributed parameters, while the Mann–Whitney *U* test was performed in non-normally distributed variables. Discontinuous parameters were calculated as a frequency and expressed as percentages. Univariate analyses between qualitative parameters were made by conducting the χ2 test and Yates’ test. We used logistic regression analyses to compute the relative risks of an endpoint correlated with different factors. Multivariate analysis to identify independent predictive factors was performed by adopting Cox’s logistic regression. The entry criterion was *p* < 0.1, and the permanence criterion was *p* < 0.05. The predictive value was expressed as RR with a 95% CI. Kaplan–Meier curves were adopted to compare survival between these two groups, and significance was assessed by a log-rank test. For all tests, significance was set at *p* < 0.05.

## 3 Results

### 3.1 Basic medical and sociodemographic characteristics

There were 653 patients in the DFU group and 510 patients in the NFU group, for a total of 1163 patients in the study sample after patients without follow-up were excluded. Regarding age, gender, HbA1c, and diabetes duration, there were substantial differences between the DFU and NFU groups. All findings are displayed in Table 1.

**TABLE 1:** Basic characteristics of participants and comparison between DFU (diabetic patients with foot ulceration) and NFU (diabetic patients without foot ulceration) groups. *p < 0.05, **p < 0.01, and ***p < 0.001.

Variable	Total (*n* = 1163)	NFU (*n* = 510)	DFU (*n* = 653)	Z-value	*p*-value	Significance (*)
Age (in years)∗	66.8 ± 11.5	65.9 ± 9.7	70.9 ± 11.7	−62.746	<0.0001	***
Gender						
Male	670 (57.5%)	271 (53.1%)	399 (60.8%)	−2.632	0.008	**
Female	496 (42.5%)	239 (46.9%)	257 (39.2%)			
HbA1c	9.2 ± 2.4	9.3 ± 2.3	9.1 ± 2.5	−3.751	<0.0001	***
Diabetes duration	11.5 ± 7.6	16.2 ± 8.6	16.8 ± 8.3	−3.964	<0.0001	***

### 3.2 Blood markers for predicting risk factors of DFU

Before receiving therapy, blood samples were taken from all patients hospitalized in our department for DFU or NFU to identify blood markers for predicting the risk of DFU. The results showed that C-reactive protein, albumin, creatinine, alkaline phosphatase, procalcitonin, platelets, 25-hydroxyvitamin D, 2-microglobulin, monocyte ratio, low-density lipoprotein cholesterol (LDL), triglyceride, alanine aminotransferase, aspartate aminotransferase, creatine kinase, and total cholesterol were significantly different between the DFU and NFU groups (Table 2).

**TABLE 2:** Comparison of risk factors between DFU (diabetic patients with foot ulceration) and NFU (diabetic patients without foot ulceration) groups. *p < 0.05, **p < 0.01, and ***p < 0.001.

Variable	NFU (*n* = 510)	DFU (*n* = 653)	Z-value	*p*-value	Significance (*)
C-reactive protein	68.7 (21.4–149.6)	77.1 (33.4–99.7)	−3.843	<0.0001	***
Albumin	43.1 (40.2–45.3)	38.3 (34.3–42.1)	−86.94	<0.0001	***
Creatinine	73.8 (56.6–137.7)	98.8 (70.4–194.4)	−65.916	<0.0001	***
Alkaline phosphatase	84 (68.0–104.0)	78.1 (60.6–109.0)	−15.090	<0.0001	***
Procalcitonin	15.9 (7.4–96.3)	200.0 (7.5–200.0)	−9.166	<0.0001	***
Platelets	224 (189–271)	236 (185–297)	−30.998	<0.0001	***
25-Hydroxyvitamin D	22.0 (17.0–25.5)	23.3 (18.4–31.0)	−7.108	<0.0001	***
β2-microglobulin	2.6 (2.0–4.5)	4.0 (2.7–9.7)	−18.542	<0.0001	***
Monocyte ratio	8.3 (7.0–10.0)	7.8 (6.3–9.9)	−16.274	<0.0001	***
Low-density lipoprotein cholesterol (LDL)	2.9 (2.2–3.5)	3.0 (2.3–3.8)	−4.373	<0.0001	***
Triglyceride	2.1 (1.3–3.6)	1.7 (1.1–2.8)	−7.617	<0.0001	***
HDL cholesterol	1.1 (0.9–1.4)	1.1 (0.9–1.3)	−1.716	0.086	NA
Alanine aminotransferase (ALT)	25.7 (15.4–49.2)	37.7 (17–124.1)	−40.486	<0.0001	***
Aminotransferase (AST)	23.2 (17.3–44.3)	30.6 (18.3–104.1)	−35.390	<0.0001	***
Creatine kinase (CK)	132 (82–252)	295 (109.3–921)	−157.444	<0.0001	***
Total cholesterol	4.6 (3.9–5.4)	4.4 (3.6–5.3)	−6.722	<0.0001	***
L-lactate dehydrogenase	180 (173–256)	195 (160–238)	−0.432	0.666	NA

### 3.3 Risk factors of DFU mortality

**FIGURE 1:**
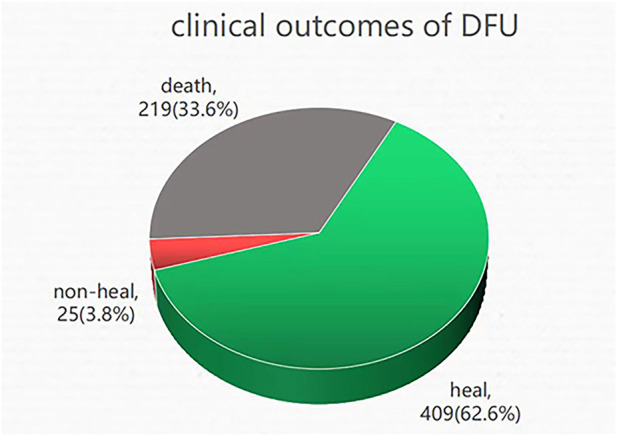
Clinical outcomes of DFU: death, non-healing, and healing. A total of 653 patients underwent successful follow-up, of which 33.6% of DFU patients lost their lives, 62.6% of DFU patients were healing, and 3.8% of DFU patients were non-healing.

In August 2022, DFU follow-up data were collected *via* telephone interview with the patient or the patient’s family. A total of 653 individuals out of 2131 were successfully followed up, while 219 DFU patients perished. These results demonstrated that 33.6% of DFU employees perished (Figure 1).

According to diabetic foot ulcer patients’ follow-up data, three types can be identified: death, healing, and non-healing. In univariate analysis, mortality correlated with advanced age (70.8 ± 12.6 vs. 67.6 ± 12.0 years, death vs. healing; *p* = 0.001), diabetes duration (11 (6–15) vs. 16 (7–21.5) years, healing vs. non-healing; *p* = 0.018), retinopathy (death vs. healing; *p* = 0.001), C-reactive protein (15.4 (4.69–45.7) vs. 8.9 (3–30.4), death vs. healing; *p* = 0.005), albumin (35.9 (30.8–39.7) vs. 38.1 (33.4–41.9), death vs. healing; *p* < 0.0001), creatinine (79.9 (60.8–112) vs. 72.7 (56.7–93.6), death vs. healing; *p* = 0.007), and procalcitonin (0.04 (0.04–0.2) vs. 0.04 (0.04–0.11), death vs. healing; *p* = 0.007) (Table 3).

**TABLE 3:** Comparison of risk factors for DFU mortality. *p < 0.05, **p < 0.01, and ***p < 0.001.

Variable	Dead (*n* = 215)	Heal (*n* = 413)	Non-heal (*n* = 25)	χ2	*p*-value
Age (in years)∗	70.8 ± 12.6	67.6 ± 12.0	70.1 ± 11.3	−3.394	0.001
Gender					
Male	135 (34%)	251 (63.2%)	11 (2.8%)	−0.492	0.623
Female	80 (31.3%)	162 (63.3%)	14 (5.5%)		
HbA1c	8 (6.8–9.5)	8.1 (6.8–9.9)	8.3 (6.6–9.2)	−0.803	0.422
Diabetes duration	10 (6–15)	11 (6–15)	16 (7–21.5)	−2.372	0.018
Diabetes type					
Type 1	1 (0.5%)	10 (2.4%)	0 (0%)	−1.772	0.076
Type 2	214 (99.5%)	403 (97.6%)	25 (100%)		
Hypertension					
Yes	149 (69.3%)	269 (65.1%)	18 (72%)	−1.05	0.294
No	66 (30.7%)	144 (34.9%)	7 (28%)		
Nephropathy					
Yes	116 (54%)	212 (51.3%)	13 (52%)	−0.624	0.533
No	99 (46%)	201 (48.7%)	12 (48%)		
Retinopathy					
Yes	93 (43.3%)	238 (57.6%)	17 (68%)	−3.42	0.001
No	122 (56.7%)	175 (42.4%)	8 (32%)		
Neuropathy					
Yes	174 (80.9%)	338 (81.8%)	20 (80%)	−0.279	0.781
No	41 (19.1%)	75 (18.2%)	5 (20%)		
C-reactive protein	15.4 (4.7–45.7)	8.9 (3–30.35)	4.9 (1.4–16.3)	−2.815	0.005
Albumin	35.9 (30.8–39.7)	38.1 (33.4–41.9)	37.9 (33.4–40.1)	−3.586	<0.0001
Creatinine	79.9 (60.8–111.6)	72.7 (56.7–93.6)	71.5 (52.0–86.9)	−2.701	0.007
Alkaline phosphatase	64.1 (52.9–85.0)	65.3 (51.8–83.1)	68.2 (58.0–100.5)	−0.542	0.588
Procalcitonin	0.04 (0.04–0.2)	0.04 (0.04–0.11)	0.04 (0.04–0.04)	−2.696	0.007
White cell count (WCC)	7.5 (5.8–10.7)	7.3 (5.8–9.5)	6.4 (5.5–7.9)	−1.251	0.211

After adjusting for age, diabetes duration, retinopathy, C-reactive protein, albumin, creatinine, and procalcitonin, a logistic regression analysis identified only age and albumin as independent predictors of death (Table 4). Higher DFU mortality was significantly associated with increasing age and decreasing albumin levels.

**TABLE 4:** Multivariate analysis of risk factors for DFU mortality. *p < 0.05, **p < 0.01, and ***p < 0.001.

Variable	β-value	T-value	95%CI	*p*-value
Age	0.117	2.878	0.002 to 0.009	0.004
Albumin	−0.091	−2.23	−0.015 to ∼−0.001	0.026
Creatinine	0.014	0.276	−0.001 to 0.001	0.783
C-reactive protein	−0.008	−0.137	−0.002 to 0.002	0.891
Procalcitonin	−0.036	–0.700	−0.007 to 0.003	0.484
Retinopathy	−0.084	−1.642	−0.208 to 0.019	0.101

### 3.4 Treatment by NPWT in combination with PRP

The treatment efficacy of NPWT alone and NPWT combined with PRP therapy for DFU was evaluated. There are two groups. The combined group was treated with NPWT first and then PRP, while the control group was treated with NPWT first and then traditional dressings. A total of 31 DFU patients meeting the inclusion criteria were recruited. There were no significant differences between these two groups concerning basic characteristics such as age, gender, HbA1c, hypertension, nephropathy, retinopathy, neuropathy, diabetes duration, C-reactive protein, and procalcitonin (Table 5). In terms of healing time, however, ulcers in the combination group (60 (1560) d) healed much faster than ulcers in the control group (91 (7298) d) (*p* < 0.0001). Quantitatively, the combined group’s 93.8% healing rate was much greater than the control group’s 53.3% healing rate. The combined group’s mortality rate of 6.3% dropped more sharply than the control group’s mortality rate of 40% (*p* = 0.011). Overall, this study indicated that, compared to NPWT paired with standard dressing, NPWT combined with PRP therapy could accelerate ulcer healing and reduce DFU mortality.

**TABLE 5:** Comparison of the combined group and control group, follow-up wound healing time, and outcome in the two groups. *p < 0.05, **p < 0.01, and ***p < 0.001.

Variable	Combined group	Control group	Z-value	*p*-value
Age (in years)∗	61.1 ± 13.5	63.5 ± 11.9	−0.020	0.984
Gender				
Male	13 (54.2%)	11 (45.8%)	−0.518	0.604
Female	3 (42.9%)	4 (57.1%)		
Hypertension				
Yes	12 (75%)	10 (66.7%)	−0.503	0.615
No	4 (25%)	5 (33.3%)		
Nephropathy				
Yes	12 (75%)	8 (53.3%)	−1.240	0.215
No	4 (25%)	7 (46.7%)		
Retinopathy				
Yes	10 (62.5%)	11 (73.3%)	−0.634	0.526
No	6 (37.5%)	4 (26.7%)		
Neuropathy				
Yes	14 (87.5%)	12 (80%)	−0.558	0.577
No	2 (12.5%)	3 (20%)		
HbA1c	9.4 ± 2.7	9.4 ± 2.1	−0.494	0.621
Diabetes duration	10.9 ± 8.7	12.2 ± 6.7	−0.654	0.513
C-reactive protein	20 (7.0–62.4)	16.8 (10.9–89.3)	−0.632	0.527
Albumin	36.1 ± 5.9	33.4 ± 7.1	−1.037	0.3
Creatinine	76.7 ± 26.4	81.8 ± 27.4	−0.353	0.724
Alkaline phosphatase	75.2 ± 23.9	107.5 ± 129.3	−0.486	0.627
Procalcitonin	0.04 (0.04–0.11)	0.17 (0.04–0.29)	−1.669	0.095
Number of leukocytes	10.9 ± 4.5	9.3 ± 8.0	−1.455	0.146
Outcome				
Heal	15 (93.8%)	8 (53.3%)	−2.542	0.011
Death	1 (6.3%)	6 (40%)		
Ulcer healing time(d)	60 (15–60)	91 (72–98)	−17.078	<0.0001

### 3.5 Typical case in the combined group

**FIGURE 2:**
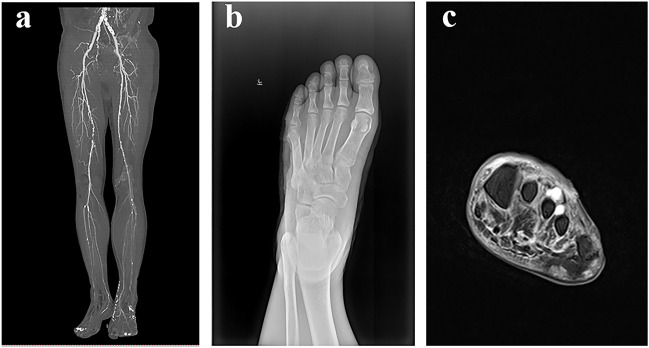
Aids to the examination. **(A)**: Below-knee CTA showed that lower limbs with arteriosclerosis and lumen had various degrees of stenosis. **(B)** Foot skew radiographs showed that the soft tissue density of the lateral left foot was non-uniform and locally absent. **(C)** Foot MRI showed that there was no obvious abnormality in the left bone marrow, plantar and dorsal regions of the foot muscle gap had a flocculently visible signal on T2, and plantar fascia thickening.

The patient, a 57-year-old man, was diagnosed with type 2 diabetes mellitus in the neighborhood hospital after exhibiting symptoms of dry mouth, polydipsia, and polyuria, 13 years prior without any causes. One week ago, the patient developed blisters on the left foot after walking for a long time. The patient’s inability to walk and his red, ulcerated, and pus-filled skin were the main reasons for his admission to the hospital. Blisters that self-cut themselves caused the left foot to swell. The patient experienced unpleasant symptoms, including nausea, chest tightness, below-knee numbness, vomiting, and pharyngeal soreness. Lower limbs had varying degrees of stenosis and arteriosclerosis, as shown by below-knee CT angiography (CTA) (Figure 2A). The soft tissue density of the lateral left foot was non-uniform and localized absent, according to foot skew radiography (Figure 2B). A foot magnetic resonance imaging (MRI) scan revealed thickening of the plantar fascia, a flocculently apparent signal in the plantar and dorsal foot muscle gaps, and no obvious abnormality in the left bone marrow (Figure 2C).

**FIGURE 3:**
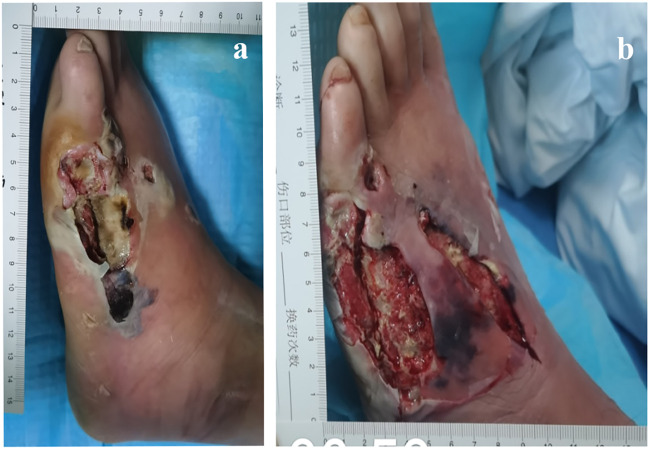
57-year-old man with diabetes mellitus had a non-healing ulcer in the front of his left foot. **(A)** After admission. **(B)** After debridement.

**FIGURE 4:**
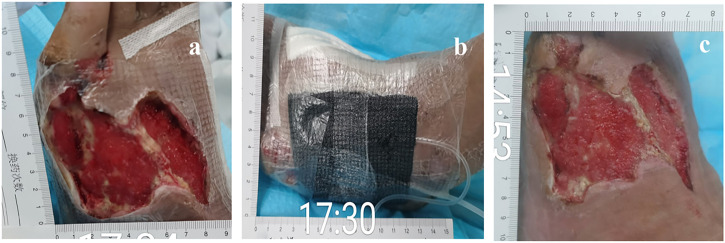
57-year-old man with diabetes mellitus had a non-healing ulcer in the front of his left foot. **(A)** Before being treated with NPWT. **(B)** During the treatment with NPWT. **(C)** After being treated with NPWT.

**FIGURE 5:**
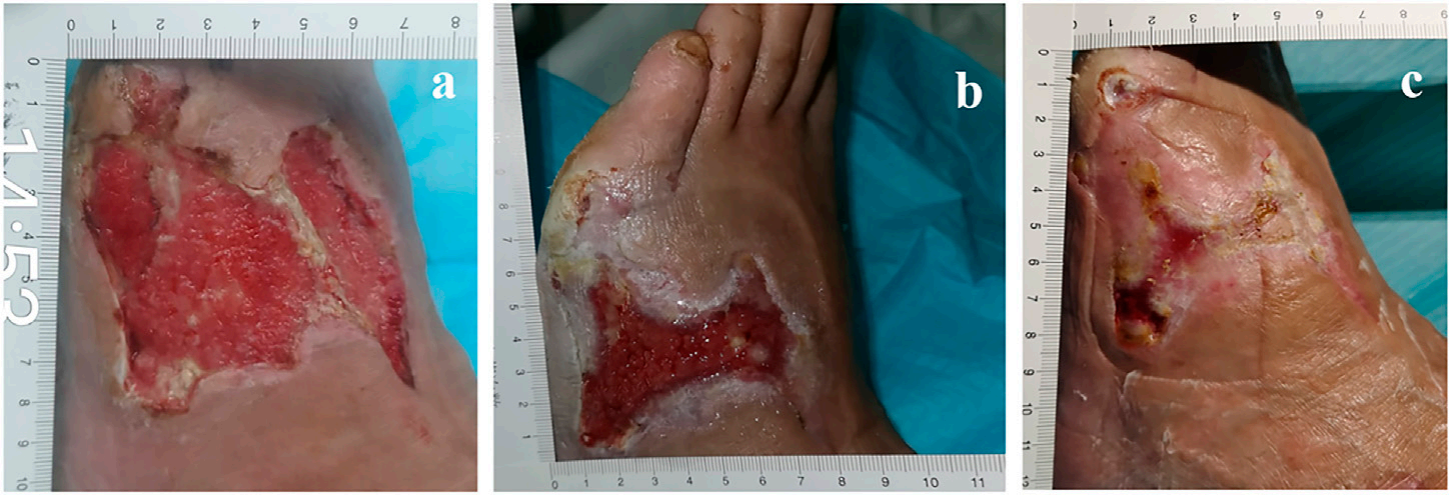
57-year-old man with diabetes mellitus had a non-healing ulcer in the front of his left foot. **(A)** Before using PRP. **(B)** After NPWT, the PRP was administered immediately. **(C)** After the PRP was administered for a second time.

After hospitalization, the patient was subjected to debridement (Figures 3A, B). On day 5, NPWT was administered as soon as the wound beds were free of apparent necrotic tissue and purulent discharge (Figures 4A–C). A controllable negative pressure environment is given by adopting NPWT to achieve wound drainage and reduce wound infection. Following NPWT, individuals in the combined group received two applications of topical PRP (Figures 5A, B). After 50 days, the patient’s wounds had fully healed.

## 4 Discussion

The earlier research assessing the risk of DFU morbidity could only account for basic sociodemographic characteristics. Few studies have been conducted on blood markers to predict the risk of DFU. In this study, we examined blood markers for predicting DFU morbidity and DFU mortality risk factors. In addition, this study reported for the first time that the therapy of combining NPWT and PRP accelerated DFU wound healing and decreased the DFU mortality rate in comparison with the treatment of NPWT alone.

Interestingly, our results showed substantial variations between DFU and NFU in blood parameters, such as C-reactive protein (CRP), procalcitonin (PCT), and platelets. CRP and PCT (Uivaraseanu et al., 2020) were the most widely used inflammation biomarkers. CRP has been reported to distinguish between grade 1 and grade 2 DFUs (Jeandrot et al., 2008). Recent research has reported that PCT may be used as a diagnostic marker in conjunction with CRP to distinguish between infected and non-infected foot ulcers (Jonaidi Jafari et al., 2014). These findings suggest grade 1 and grade 2 DFU groups might be distinguished by increased CRP and PCT in infectious DFU patients. Moreover, the present study showed that the blood ALT and AST levels of DFU patients were significantly higher than those of NFU patients. Kim and Han (2018) found that AST and ALT independently predicted the future development of metabolic syndrome. Relevant studies have shown a high correlation between metabolic syndrome prevalence and increased AST or ALT levels (Seo et al., 2022). Our research suggests DFU and metabolic syndrome may be connected when taken as a whole. Our study also identified several distinct risk factors for DFU mortality, including albumin and age by logistic regression analysis, offering new risk factors for DFU mortality.

NPWT has emerged as a crucial adjuvant therapy method for the care of diabetic foot wounds (Liu et al., 2018). Numerous clinical randomized controlled trials have demonstrated that NPWT can significantly enhance the wound healing rate, shorten the wound healing time, and lower the amputation rate compared to typical diabetic foot wound treatment (Mohseni et al., 2019). However, the treatment effects of combined NPWT and PRP on DFU have remained unexplored. Notably, the combination of NPWT and PRP expedited wound healing and decreased mortality in our study. Due to the risk of immunological reactions and cross-contamination, allogeneic platelet-rich plasma is currently underutilized in therapeutic settings (Mastrogiacomo et al., 2022). In our study, patients treated with PRP exhibited no signs of local inflammation, allergies, or other adverse responses.

Our study also showed that after receiving NPWT combined with PRP treatment, blood parameters, such as CRP, PCT, creatinine, albumin, and platelets, significantly changed in DFU (Table 6). The likelihood of a second foot ulcer following the first ulcer ranges from 30% to 87% (Skafjeld et al., 2015). Previously, inflammation-driven epigenetic alterations that were retained after restoration to a normoglycemic environment also contributed to altered cell activity in DFU (Cao et al., 2022). This phenomenon, named “hyperglycemic memory,” likely has a major impact on the high risk of recurrence of diabetic ulcers. In the “hyperglycemic memory” phenomenon, platelet count was upregulated (Tokarz-Deptula et al., 2021). In addition, CRP and PCT have been employed as inflammation-related infection biomarkers (Cao et al., 2022). Previous investigations have shown that one of the independent risk variables associated with a DFU recurrence was increased CRP (Dubsky et al., 2013). These results raised the prospect of NPWT combined with PRP therapy, which might improve blood parameter normalization and lower the recurrence rate of diabetic foot lowering “hyperglycemic memory,” particularly by CPR, and considerably lower PCT.

**TABLE 6:** Significant difference was observed between admission and discharge in diabetic patients after treatment with NPWT and PRP. *p < 0.05, **p < 0.01, and ***p < 0.001. PLT: patients of blood parameters, treated with NPWT combined with PRP.

Variable	DFU in admission PLT	DFU in discharge PLT	Z-value	*p*-value
C-reactive protein	77.1 (33.4–99.7)	43.7 (15.1–84.8)	−37.573	<0.0001
Albumin	37.6 (33.3–41.1)	38.3 (34.3–42.1)	−12.308	<0.0001
Creatinine	98.8 (70.4–194.4)	95 (68.4–173.2)	−10.668	<0.0001
Procalcitonin	200.0 (7.5–200)	0.9 (0.1–5.2)	−9.833	<0.0001
Platelets	236.0 (185–297)	239.0 (190.0–305.0)	−15.664	<0.0001

However, the patient-related factors (such as compliance, offloading, microvascular state, neuropathy, and nutritional status) were not covered in this study, which may impact the chosen long-term clinical outcomes.

## 5 Conclusion

Our study demonstrates that blood markers, such as CRP, PCT, AST, and ALT, can be utilized to predict DFU morbidity risk factors. Only albumin and age were revealed to be independent DFU mortality predictors. According to the findings of the present study, patients who received NPWT in conjunction with PRP observed quicker wound healing and a decreased mortality rate. Collectively, our findings identified unique risk factors for morbidity and mortality associated with DFU, and they could lead to a practical, efficient, and secure biological therapy as a ready-to-use treatment for DFU consistent with the current pilot trial.

## Data Availability

The original contributions presented in the study are included in the article/Supplementary Materials; further inquiries can be directed to the corresponding author.
